# A Case Series of Eye Removal Surgeries: Five Years of Experience at a Tertiary Eye Care Centre in Malaysia

**DOI:** 10.7759/cureus.65986

**Published:** 2024-08-02

**Authors:** Lhacha Wangdi, Niki Ho Wai Wye, Yong Meng Hsien, Ainal Adlin Naffi, Mae-Lynn Catherine Bastion

**Affiliations:** 1 Department of Ophthalmology, Gyalyum Kesang Choden Wangchuck National Eye Centre, Thimphu, BTN; 2 Department of Ophthalmology, Hospital Universiti Kebangsaan Malaysia, Kuala Lumpur, MYS; 3 Department of Ophthalmology, Hospital Canselor Tuanku Muhriz (HCTM) Universiti Kebangsaan Malaysia, Kuala Lumpur, MYS; 4 Department of Ophthalmology, Universiti Kebangsaan Malaysia Medical Center, Kuala Lumpur, MYS

**Keywords:** exenteration, enucleation, evisceration, anophthalmic surgery, eye removal surgery

## Abstract

Background: Eye removal surgeries, also called anophthalmic surgeries, are usually performed for a painful blinded eye due to various underlying causes. In this case review, we intended to study the indications, the types of anophthalmic surgeries, and post-operative complications related to eye removal surgeries.

Method: Five years of retrospective case review of surgical eye removals was conducted from 1st June 2018 to 31st May 2023 at Hospital Canselor Tuanku Muhriz (HCTM), University of Kebangsaan Malaysia (UKM). Medical record files were used to analyse the age, gender, affected eye, types of surgeries, and indications of the eye removal surgery.

Results: Fourteen eyes underwent anophthalmic surgeries inclusive of evisceration (78.57%, n = 11), enucleation (14.29%, n = 2), and exenteration (7.14%, n = 1). Among the evisceration group, 63.64% (n = 7) were due to endophthalmitis, 27.27% (n = 3) were due to ocular trauma, and 9.09% (n = 1) were done for a painful blind due to neovascular glaucoma. Two enucleation surgeries were performed for retinoblastoma and one exenteration for orbital metastatic malignancy.

Conclusion: The preferred choice of anophthalmic surgery was in favour of evisceration, especially when the underlying causes were due to benign conditions. The most common indications of anophthalmic surgeries were endophthalmitis, trauma, and malignancies. Enucleation and exenteration were performed mainly for the blinded eye due to the intraocular malignancies and malignancy with an extraocular spread. A fairly lesser number of anophthalmic surgeries over the five years could imply an improvement in the conservative management approach of a painful blinded eye.

## Introduction

Some of the eye conditions warrant the surgical removal of the eye itself. The removal of the eye is usually performed as a therapeutic treatment for an intractable painful blind eye or as an ongoing diagnostic assessment of an eye. Eye removal surgery is also called anophthalmic surgery. The surgical goal of anophthalmic eye surgery is to make the patient pain-free and ensure a reasonable cosmetic outcome for a painful blind eye [1]. During the anophthalmic surgery, the damaged eye will be removed and the surgical lost volume will be replaced [2]. The cosmetic appearance will be achieved by using an ocular prosthesis a month after the surgery.

The removal of the eye involves either the removal of the contents of an eye or the entire eye globe with or without the removal of the orbital contents. There are three surgical procedures for the surgical removal of an eye, which are evisceration, enucleation, and exenteration. The surgical principle of evisceration includes the removal of the contents of an eye leaving behind the intact scleral shell and optic nerve, whereas, in enucleation, the entire eye globe, including the scleral shell and a part of the optic nerve, is removed [3]. Exenteration is an extended surgical procedure that involves the removal of the eye globe along with the orbital contents [4].

There are various indications implicated for an anophthalmic eye surgery; however, it is mostly performed as a last resort of surgical management for a painful blind eye [1,2,5]. Among the evisceration group, the most commonly reported indications are painful blinded eyes due to endophthalmitis, ocular trauma, neovascular glaucoma, phthisis, and cosmetic reasons [6].

In Malaysia, there is no reported case series of eye removal surgeries. This case series was conducted to analyse the demographic profile of patients and to review the common indications and surgical outcomes of eye removal surgeries in a tertiary eye care setting.

## Materials and methods

This study was conducted as a hospital-based study of eye removal surgeries performed in the eye care centre of Hospital Canselor Tuanku Muhriz (HCTM) of the University of Kebangsaan Malaysia (UKM). HCTM is a semi-government tertiary eye care centre in Malaysia that provides special eye care services. The study was conducted in both the outpatient and inpatient departments of HCTM. The main objective of this study was to review the demographic profile, indications, and surgical outcomes of patients who underwent eye removal surgeries.

The study was designed as a retrospective study. The cases of eye removal surgery were collected for the past years from 1st June 2018 to 31st May 2023. All patients who underwent eye removal surgeries were included in this study irrespective of age group and gender. Those patients who did not give consent for the use of their clinical information for the study were excluded. This study also excluded other eye-sparing surgeries and vision restorative eye surgeries. Verbal consent was obtained from the patients by phone to use their old recorded clinical information for the study. A convenient sampling method was used to review the clinical details of patients retrospectively. The patients’ record files and electronic medical records were used to review the clinical information.

In this study, the clinical information assessed included the clinical profile of the patients undergoing eye removal surgery. We looked at the indications of eye removal surgeries, pre-operative preparations, including consenting, intra-operative procedures, and post-operative care. In each case, we reviewed the pre-operative diagnosis of the eye conditions followed by a management plan. Intra-operatively, we look at the types of eye surgeries done and their surgical procedures. Post-operatively, we reviewed the post-operative management and follow-up plan for each case. We also looked at the timeframe of the use of ocular prosthesis after the eye removal surgery. Any complications and their management both intra-operatively and post-operatively were complied with.

Data compilation included age, gender, eye affected, indications, and the types of surgeries and the complications of the eye removal surgeries. Data were recorded in an Excel sheet (Microsoft Corporation, Redmond, WA) and the statistical analysis was done using SPSS version 20 (IBM Corp., Armonk, NY). Statistical analysis was done in the form of percentages for the common age group, gender, common indications, and types of eye removal surgeries.

## Results

**Figure 1:**
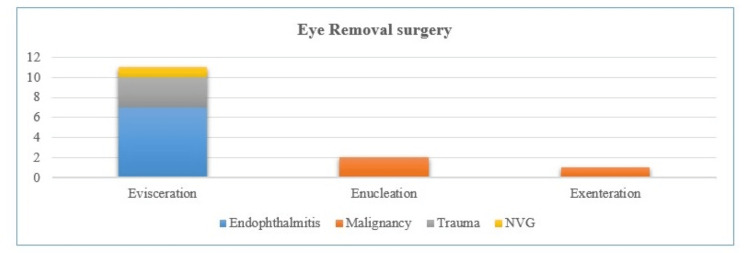
Types and indications of anophthalmic surgeries performed in Hospital Canselor Tuanku Muhriz (HCTM), University of Kebangsaan Malaysia (UKM) from 1st June 2018 to 31st May 2023: evisceration (n = 11), enucleation (n = 2), and exenteration (n = 1). NVG: neovascular glaucoma.

In the clinical case review, all the patients who underwent eye removal surgeries were initially assessed for the underlying causes and the indications for the eye removal surgery were discussed with patients. Operated eyes were either painful blinded eyes or eyes with an untreatable ocular malignancy with poor visual potential. Based on the underlying causes, the patient either underwent evisceration, enucleation, or exenteration (Figure 1). Following eye removal surgery, routine care, including topical steroids and antibiotics, was provided. They were followed in one week and four weeks post-operatively. During follow-up, any post-operative complications encountered were treated appropriately.

Among the evisceration group, the commonest indication was endophthalmitis (63.64%, n = 7), followed by ocular trauma (27.27%, n = 3). Out of seven cases of endophthalmitis, five had endogenous endophthalmitis and one each of post-operative endophthalmitis and exogenous endophthalmitis. Two out of three cases of ocular trauma requiring evisceration were due to severe blunt trauma, one had an open globe injury leading to a phthisis. Enucleation surgeries were performed mainly for the intraocular malignancy, both the enucleated cases had retinoblastoma. Among the eye removal surgeries, only one case had exenteration where a lid-sparing procedure was performed for metastatic renal adenocarcinoma with intra-orbital and intra-ocular spread.

**Figure 2:**
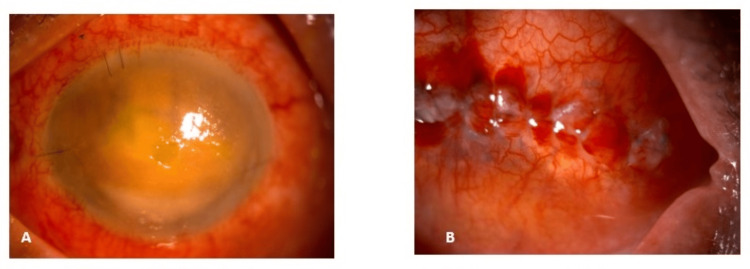
A 76-year-old lady had right eye post-operative endophthalmitis a month following phacoemulsification. (A) Right eye showing intense chemosis, diffuse cornea oedema, and intraocular abscess. (B) Two weeks after evisceration with a 20 mm orbital implant showing wound healing with granulation tissue.

**Figure 3:**
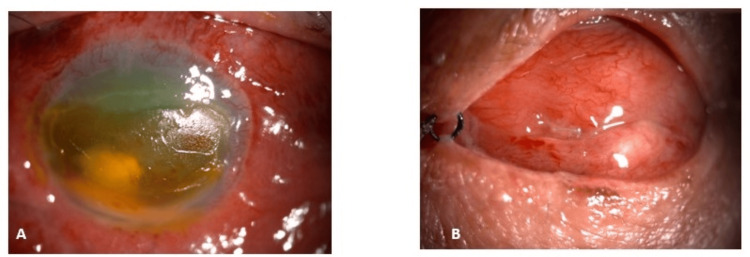
An 86-year-old female had a right eye cornea ulcer complicated with endophthalmitis. (A) Right eye showing chemosis, near total cornea dry epithelial defect, cornea oedema, and blood-stained hypopyon. (B) A month after evisceration of the same eye showing a well-healed wound.

Although the eye removal surgeries were performed by different surgeons, similar standard surgical steps were followed for all the cases; however, the use of intra-operative orbital implants was not uniform among eviscerated and enucleated eyes. Among the eviscerated eyes, only five eyes out of 11 eyes had used an orbital implant; on the other hand, both the enucleated eyes had used the orbital implants. Once all the surgical wounds were healed and healthy, ocular prostheses were provided one month after the surgery. The majority of the anophthalmic surgeries had uneventful outcomes. Only two cases were complicated with post-operative wound dehiscence, which was restored successfully. Figures 2, 3 are examples of case descriptions of successful eye removal surgeries performed in HCTM.

## Discussion

Generally, anophthalmic surgeries are performed mainly for end-stage painful blinded eyes due to various underlying causes [1,2]. Globally, among the anophthalmic surgeries, evisceration is a preferred surgical choice mainly because it achieves better functional mobility of a prosthetic eye shell and therefore it has a better cosmetic result than enucleation or exenteration [7,8]. However, during evisceration, there are more surgical manipulations of intraocular contents and therefore, there is a theoretical risk of seeding intraocular malignancy. Hence, evisceration is not a choice of surgery when the intraocular malignancy is the cause of the painful blinded eye [9].

In this case series, out of 14 anophthalmic surgeries, 78.57% (n = 11) underwent evisceration and all the eviscerations were performed only for the benign underlying causes, whereas two cases of enucleation were performed for the intraocular malignancy without the extraocular spread while only one exenteration was performed for metastatic tumour with orbital spread. These findings were consistent with previous similar studies where evisceration was reported as the commonest anophthalmic surgery, while enucleation surgeries were performed for the blinded eye due to intraocular malignancies, and exenteration surgeries were performed for advanced malignancy with orbital spread [2,10,11].

The commonest indication of evisceration in this case review was endophthalmitis (63.64%, n = 7), followed by ocular trauma (27.27%, n = 3). Endophthalmitis was reported as one of the commonest indications of evisceration, which implies the fulminant and aggressive nature of endophthalmitis requiring eye removal [12]. Looking at the total number of anophthalmic surgeries over the five years from 1st June 2018 to 31st May 2023 at HCTM, UKM, there were only 14 reported cases of anophthalmic surgeries. The number of anophthalmic surgeries performed in this case review was very less compared to other similar previous anophthalmic surgery case reviews (Table 1). For example, 68 eyes of 67 patients underwent enucleation or evisceration at Jordan University Hospital between August 2006 and June 2011. In another case review in a tertiary eye centre in Calabar, Nigeria, a total of 137 eyes were surgically removed over 10 years [5,10]. Over the years, fewer cases of anophthalmic surgeries could imply a more conservative approach to eye-sparing management or advancement in eye care services in tertiary eye care centres in Malaysia.

**Table 1:** Comparative series of retrospective case reviews of surgical removal of the eye.

Surgical removal of eyes	Current case series, % (total)	Case series by Mpyet et al. [10], % (total)	Case series by Kagmeni et al. [1], % (total)
(1a) Evisceration	78.7 (11)	88.3 (91)	71.54 (181)
(1b) Enucleation	14.29 (2)	7.8 (8)	27.27 (69)
(1c) Exenteration	7.14 (1)	3.9 (4)	1.18 (3)
(2) Common indications	Endophthalmitis: 50 (7)	Ruptured globe: 45.6 (47)	Perforated corneal ulcer: 33.2 (84)
	Malignancy: 21.43 (3)	Intractable infection: 34.0 (35)	Endophthalmitis: 18.2 (46)
	Trauma: 21.43 (3)	Unsightly blind eye: 6.8 (7)	Trauma: 17.4 (44)
	Invasive orbital malignancy: 7.14 (1)	Painful blind eye: 6.8 (7)	Painful blind eye: 11.5 (29)

One of the dreaded but a very rare post-operative complication of eye removal surgery is sympathetic ophthalmia (SO). There are few rare reports of SO following an evisceration [13,14]. However, SO is due to multifactorial causes. Although there is a plausible relation with evisceration, there is no concrete proof of evidence for eye removal surgery as a true risk factor of SO [15]. In this case review, there were no reported incidences of SO following eye removal surgeries. In this case series, the most common post-operative complication of eye removal surgeries was wound dehiscence.

This study is a hospital-based retrospective study, therefore the overall findings pertinent to this study are not the true representative of eye removal surgeries in Malaysia. Due to the retrospective nature of the study, which was based on the clinical information from the medical record files, there could be missing data. Further large-scale prospective research on eye removal surgery in Malaysia is needed to get the true picture of indication, current surgical practices, and surgical outcomes.

## Conclusions

The preferred trend of anophthalmic surgery in the tertiary eye care centre was in favour of evisceration over enucleation or exenteration, especially when the underlying cause included benign conditions. There were no reported incidences of sympathetic ophthalmia following evisceration. Enucleation was performed mainly for the blinded eye due to the intraocular malignancies and the exenteration for the malignancy with an extraocular spread. Compared with the international case reports, lesser cases of anophthalmic surgeries in the tertiary eye care hospital in Malaysia necessitates further research to study the possible reasons for the probable decline in the rate of eye removal surgeries.
